# Watching the human retina breath in real time and the slowing of mitochondrial respiration with age

**DOI:** 10.1038/s41598-023-32897-7

**Published:** 2023-04-20

**Authors:** Pardis Kaynezhad, Ilias Tachtsidis, Sobha Sivaprasad, Glen Jeffery

**Affiliations:** 1 Institute of Ophthalmology, University College London, 11-43 Bath St, London, EC1V9EL UK; 2 Department of Medical Physics and Biomedical Engineering, University College London, Gower St, London, WC1E6BT UK

**Keywords:** Visual system, Optics and photonics

## Abstract

The retina has the greatest metabolic demand in the body particularly in dark adaptation when its sensitivity is enhanced. This requires elevated level of perfusion to sustain mitochondrial activity. However, mitochondrial performance declines with age leading to reduced adaptive ability. We assessed human retina metabolism in vivo using broad band near-infrared spectroscopy (bNIRS), which records colour changes in mitochondria and blood as retinal metabolism shifts in response to changes in environmental luminance. We demonstrate a significant sustained rise in mitochondrial oxidative metabolism in the first 3 min of darkness in subjects under 50 years old. This was not seen in those over 50 years. Choroidal oxygenation declines in < 50 s as mitochondrial metabolism increases, but gradually rises in the > 50 s. Significant group differences in blood oxygenation are apparent in the first 6 min, consistent with mitochondrial demand leading hemodynamic changes. A greater coupling between mitochondrial oxidative metabolism with hemodynamics is revealed in subjects older than 50, possibly due to reduced capacity in the older retina. Rapid in vivo assessment of retinal metabolism with bNIRS provides a route to understanding fundamental physiology and early identification of retinal disease before pathology is established.

## Introduction

The retina is unique in having the greatest metabolic demand in the body^1–3^ with significant dynamic changes in metabolism required to maintain visual function over an almost 9 log unit luminance range. This is particularly true in dark adaptation when rods are more active and depolarised as retinal sensitivity increases^4–6^. Moreover, the retina unlike any other organ can be visualised directly through the transparent lens, offering the potential ability to monitor its metabolism in vivo and in real time. This offers major advantages in our understanding of fundamental issues in physiology and provides interventionist opportunities in disease progression before pathology becomes otherwise apparent. The significance of this is brought into focus in an ageing population where metabolic deterioration is common and is associated with progressive mitochondrial decline^7,8^. This in turn is associated with an increased prevalence of retinal diseases with metabolic features including diabetic retinopathy and age-related macular degeneration (AMD)^9–12^.

It is also possible to view directly changes in mitochondrial respiration, oxygenated and deoxygenated blood using broadband near-infrared spectroscopy (or bNIRS) because shifts in oxygenation and mitochondrial metabolism are associated with NIR contrast changes. bNIRS measures concentration changes in oxidized cytochrome-c-oxidase (Δ[oxCCO]), the terminal enzyme in the mitochondrial electron transport chain (ETC), which catalyses over 95% of oxygen metabolism, simultaneously with blood oxygenation and hemodynamics, through measuring changes in concentration of oxygenated and deoxygenated hemoglobin (Δ[HbO_2_] and Δ[HHb])^13–15^. We have established this technology examining spectral changes in back reflected light from the retina to measure retinal tissue oxygenation and metabolism in rodents^16,17^.

In this study, we translate this research and use bNIRS in a non-contact real time investigation into the changes in human retinal metabolism and hemodynamics revealed in reflected light when subjects are in the initial stages of dark adaptation. Here mitochondria are stressed because of the increased demand for adenosine triphosphate (ATP) to support changes in retinal sensitivity^2,4^. Dark adaptation is a standard metric for visual function assessment and reveals abnormalities in ageing that can be a precursor to retinal disease^18–20^. However, the physiological and metabolic platform of this has never been revealed. Our hypothesis is that the bNIRS-measured oxCCO, a marker for mitochondrial oxidative metabolism, is reflective of photoreceptors’ functional abilities, particularly rods, in darkness and provides a real time view of fundamental physiological dynamics that underpin visual function. Hence, we propose that there will be shifts in the oxCCO signal reflecting changing mitochondrial function in dark adaptation, and this may differ with age. We will also examine potential changes in hemodynamics and their relationship with mitochondrial metabolism as this may be compromised with age.

To achieve this, we examine the dynamics of metabolic signals embedded in light reflected back from the macular in subjects during dark adaptation. This provides a changing metabolic readout from the retinal region containing the greatest photoreceptor density with a very high mitochondrial content. As this is a largely avascular region the hemodynamic changes recorded largely arise from the choroid that provides the blood supply to the outer retina which feeds the photoreceptor population.

## Methods

All experimental protocols were performed in accordance with relevant guidelines and approved by University College London UK research ethics committee (21873/001). They were in accordance with the declaration of Helsinki. Each participant provided written informed consent prior to taking part in the study. Thirty consenting healthy subjects age between 20 and 70 years old participated in the study, 15 females and 15 males.

Subjects’ dominant eye was dilated using 1% Tropicamide (Bausch & Lomb Inc.) to standardise the degree of dilation. They were given optically clear glasses to protect their eyes from any contact with the measuring probes and seated with a stabilized head position against a head strap and a chin rest facing into a ganzfeld bowl where background illumination was tightly controlled. Two identical optical fibers (0.6 mm core diameter and numerical aperture; 0.37) were positioned in front of the eye with ~ 30° converging separation between them such that the tips were approximately 1–1.5 mm apart. The numerical aperture of both source and detector fibers was 0.37. The miniature system used here has been described previously^16,21^. Briefly, light from a miniature low power broadband light source was filtered (cut off < 710 nm) and dim NIR light was with average power < 6mW across all wavelengths was delivered to the eye through the source fiber. The detector probe collected the back-reflected light. This was transmitted to a miniature spectrometer and then to a PC for data processing using wavelengths between 780 and 900 nm and the UCLn algorithm, based on the Modified Beer-Lambert Law^22,23^. Relative changes in concentration of HHb, HbO_2_, and oxCCO as well as two other metrics for blood volume and oxygenation (total and difference hemoglobin: Δ[HbT] = Δ[HbO_2_] + Δ[HHb] and Δ[HbDiff] = Δ[HbO_2_]-Δ[HHb], respectively) were calculated with respect to time zero and displayed and saved with a sampling rate of 10 Hz. Residual analysis was carried out (provided in the Supplementary Material) in order to validate the measurement of oxCCO and to evaluate the goodness of the fit of the chromophore spectra to the measured attenuation^14^.

Experiments were undertaken in dark for 15 min. All subjects were exposed to uniform light inside the ganzfeld bowl with an intensity of 30 cd/m^2^ for 15 min prior to the experiment. No attempt was made to control for subjects’ head and eye movements, but they were asked to keep the dim red light representing the light source in the center of their lower visual field. Subjects reported seeing a relatively small discrete and dim central light source that could be retained in the central visual filed throughout the experiment. This would place the collecting light probe targeted towards the central macular region. Hence, the collecting probe would sample across the regions spanning peaks in rod and cone density. As the macular is around $\pm$ 8° and normal saccadic eye movements without changes in head orientation are significantly less than 20°, sampling occurred across the macular. Both probes were aligned well within the area of the fully dilated pupil.

All computational analysis were carried out in MATLAB 2018b and Microsoft Excel. Signals were smoothed using Savitzky-Golay filter to remove random spikes and baseline shifts likely induced by small eye movements and blinking. A 3rd order polynomial and a 51-points smooth span (approximately every 5 s, considering a data acquisition time of 100 ms) were found to remove high frequency noise without sacrificing the main features of the signals.

In order to estimate and compare the speed of mitochondrial metabolism and retinal oxygenation during dark adaptation, we calculated the gradients of the oxCCO and HbDiff signals (or rate of change in the concentration of oxCCO and HbDiff) at different time points of the measurement. Rate of change in the concentration was estimated with respect to time 0 according to the following equation, where [chromophore] refers to the concentration of each bNIRS metric; HHb, HbO_2_, HbT, HbDiff and oxCCO, t is the time point at which the rate of change is calculated and t0 is time zero at the start of the measurement. All measurements of concentration are expressed in micromolar x cm (μM.cm) as the pathlength of the light within the eye is unknown.

$$ {\text{Rate\, of\,  change }}\left( {{\text{RoC}}} \right)\, = \,{\text{d}}\left[ {{\text{Chromophore}}} \right]/{\text{dt}}\, = \,\left( {\left[ {{\text{Chromophore}}} \right]{\text{t }} - \left[ {{\text{Chromophore}}} \right]{\text{t}}0} \right) \, / \, ({\text{t}} - {\text{t}}0)(\upmu {\text{M cm}}/{\text{min}}). $$

Rate of change estimation provides us with two metrics; first its magnitude shows how fast these changes occur per minute (which could be regarded as the speed of mitochondrial metabolism or retinal oxygenation), secondly its sign signifies whether there is an increase (a positive RoC) or decline (a negative) in mitochondrial metabolism and retinal oxygenation. Subject data were divided into two groups < 50 (N = 18) and > 50 (N = 12) years old as clear thickening of Bruch’s membrane has been reported in subjects older than 45^35^ and there appeared to be a clear break in the data between these two age groups. RoC for each subject was estimated at t = 15 min and data were averaged and compared between the young and old group. In order to identify the earliest time from the start of dark adaptation when there is a clear distinction between subjects younger and older than 50 years old, mean rate of change for each subject was estimated from t = 1 min progressively every minute until the statistical significance was achieved.

Continuous wavelet-based semblance analysis was used to investigate the association between blood and mitochondrial signals during dark. Semblance is a measure of similarity between two signals and is the cosine of instantaneous phase difference between them. A semblance of + 1 signifies that the signals are perfectly in phase and a semblance of − 1 shows that they are perfectly antiphase^24,25^. Nonparametric Mann-Witney U test was used to compare the means and statistical significance was considered α < 0.05.

## Results

In the dark, the rate of change in oxCCO, a marker for mitochondrial oxidative metabolism, is significantly faster in younger subjects than the older subjects (< 50 vs > 50 years old).

**Figure 1:**
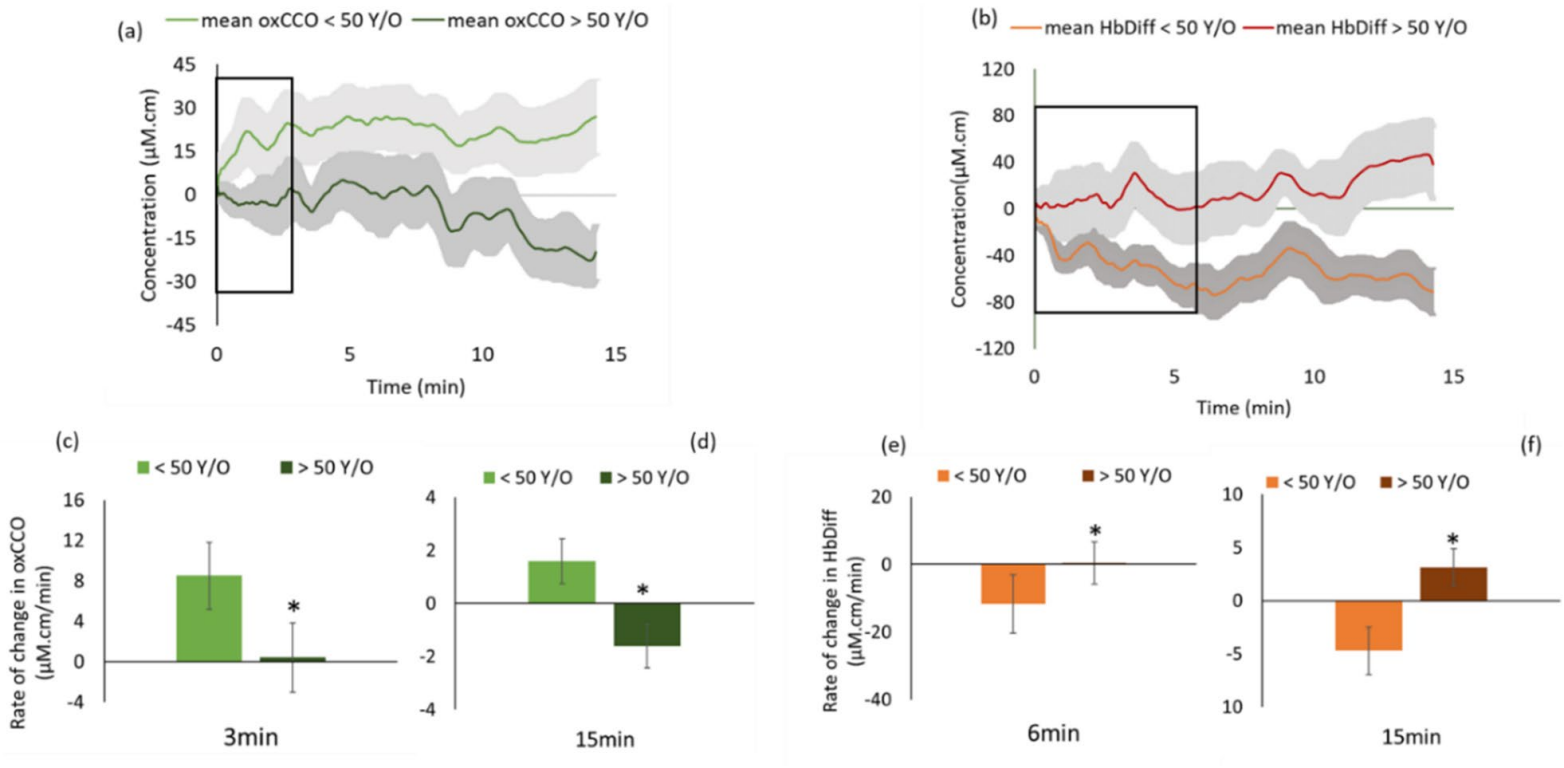
Retinal performance during dark adaptation is faster in subjects younger than 50 years old (y/o) compared to those older than 50 y/o. (**a**) Rate of oxidation of CCO in all subjects < 50 y/o is significantly greater than that for over 50 s as early as 3 min after dark onset. (**b**) Group data representing mean rate of oxidation of CCO in younger (n = 18) and older groups (n = 12). Oxidative metabolism is ~ 8 times higher in under 50 s within 3 min of dark adaptation. (**c**) Rate of change in retinal oxygenation (HbDiff = HbO_2_-HHb) is significantly greater in adults < 50 y/o during the first 6 min of dark adaptation (steeper gradient in < 50 y/o). Retinal oxygenation drops in in the younger subjects because of increased mitochondrial metabolism in dark as seen in (**a**), leading to increased O_2_ uptake by the mitochondria hence increasing the amount of deoxygenated blood (HHb). In older subjects HbDiff is almost unchanged up to 6 min (corresponding to a stable oxCCO during the first 6 min in (**a**) and gradually builds up following a reduction in oxCCO (**a**). Solid lines in (**a,c**) are mean data for all the subjects in each group, N = 18 under 50 s and N = 12 over 50 s). Shaded areas in (**a,c**) are standard error from the mean. Pooled data are given for 3 and 15 min (**c–f**) for each metric. Error bars are standard error from the mean and asterisks signify statistical significance α < 0.05.

Figure 1a shows the average data for rate of change in oxidation of cytochrome-c-oxidase (oxCCO) in the two subject groups along with standard errors in the shaded region. At the onset of darkness, oxCCO increased in under 50 s. However, there was no change in the older age group, creating a clear separation over time in the mean oxCCO signal between young and old that was statistically significant from 3 min. This separation increased further and the rate of oxidation of CCO (d[oxCCO]/dt) was markedly greater in the younger subjects throughout the experiment. At the end of 15 min dark adaptation period, the mean concentration change of oxCCO was significantly elevated in the younger subjects (27 ± 13 µm.cm) while that in the older declined ( −20 ± 10 µm.cm).

Figure 1b shows the average data for changes in retinal hemoglobin difference (Δ[HbDiff] = Δ[HbO_2_] − Δ[HHb]) which is a marker for choroidal oxygenation in younger and older subjects. These closely mirror patterns found for oxCCO, implying that an increase/decrease in oxCCO aligns with a drop/rise in retinal oxygenation. In the younger subjects, the HbDiff drop during the measurements presumably results from photoreceptors working harder to meet the increased mitochondrial demand, hence using more of the oxygenated blood (HbO_2_) and producing more deoxygenated blood (HHb). This results in reduced choroidal oxygenation (HbDiff). In the older subjects, the concentration of HbDiff remains unchanged for the first few minutes and then gradually increases over the course of the experiment. This signifies a decline in O_2_ uptake by mitochondria in older subjects, which leads to reduced HHb and elevated HbO_2_ and hence increased HbDiff. The difference between hemoglobin difference dynamics in young and old becomes statistically significant from 6 min from start of darkness. At the end of 15 min, the average HbDiff signal was significantly reduced in the younger subjects (− 70 ± 31 µm cm) compared to the older group (40 ± 20 µm cm).

Group data for the rate of mitochondrial metabolism in young and old subjects are given in Fig. 1c,d. Histograms here represent the rate of oxCCO, in dark in the younger subjects (N = 18) compared with the older subjects (N = 12) as soon as they become statistically significant (3 min) and at the end of the measurement period (15 min). In younger subjects there is a mean increase of 8 ± 3 µm cm/min during 3 min of dark adaption compared with 0 ± 3 µm cm/min in older subjects (p = 0.03), consistent with slower mitochondrial metabolism in the older subjects. At the end of the measurement, the rate of CCO oxidation slows down in the younger subjects while CCO becomes more reduced (shown by a drop in oxCCO) in the older subjects (2 ± 1 and − 2 ± 1 µm cm/min, respectively).

Group data for aged changes in retinal oxygenation are shown in Fig. 1e,f and mirror those found for group data for mitochondrial respiration. Here, the rate of change in retinal oxygenation is significantly greater in the younger group (−12 ± 5 µm cm/min), compared to the older group (0 ± 4 µm cm/min), in the first 6 min of dark adaptation. The rapid drop in retinal oxygenation during the first 6 min of darkness, together with the greater increase in Δ[oxCCO] shown in c, is indicative of faster mitochondrial metabolism in the younger group. No significant alterations in Δ[HbDiff] during the first 6 min of dark adaptation in the older subjects (Fig. 1b,e) follows the pattern for Δ[oxCCO], as there is little change is mitochondrial oxidation during that period (Fig. 1a,c). At the end of the measurement, the difference in retinal oxygenation rate between young and old groups persists and the faster increase in HbDiff in the older subjects is compatible with greater drop in oxCCO in this group at 15 min (Fig. 1f,d, respectively). We observed no difference between retinal blood volume changes (Δ[HbT]) in dark between young and old groups, which further reinforces the fact that changes in [HbDiff] are due to changes in mitochondrial O_2_ uptake rather than changes in total blood volume.

**Figure 2:**
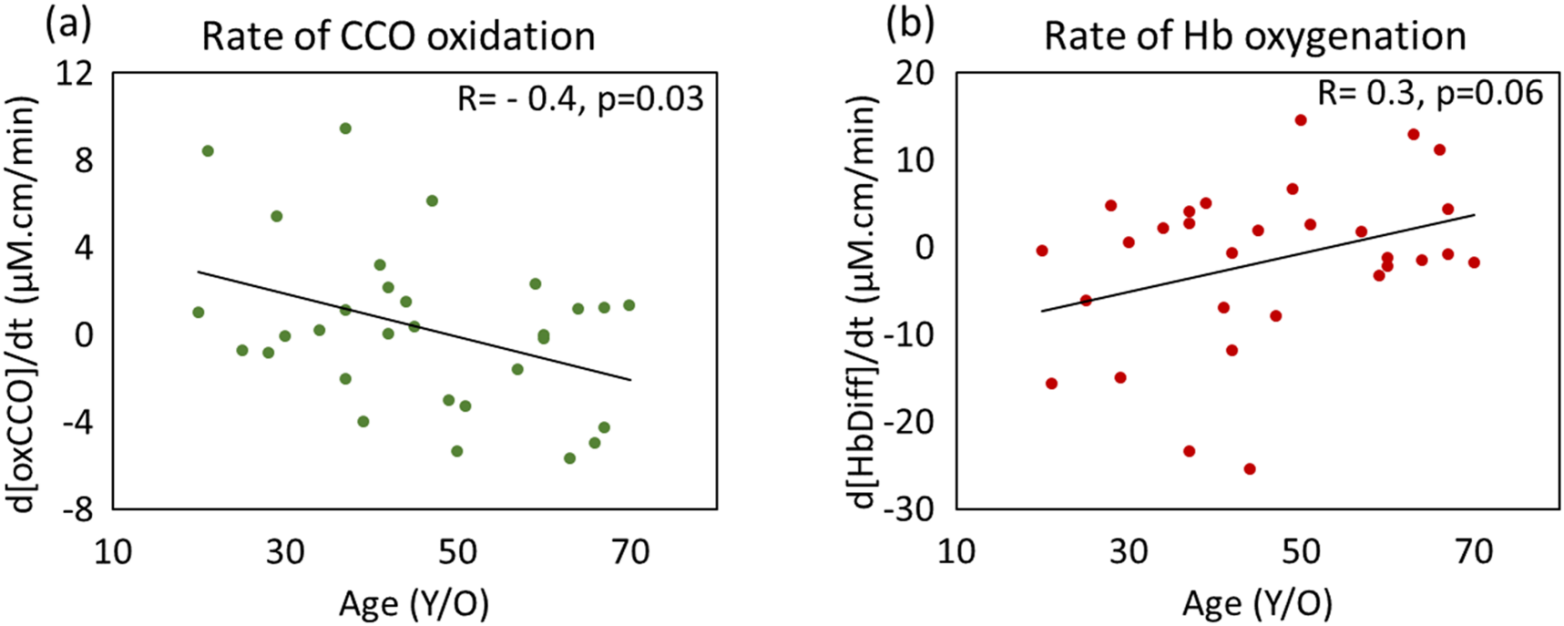
Rate of mitochondrial oxidation (**a**) and retinal oxygenation (**b**) measured at t = 15 min for all the subjects with age (N = 30). (**a**) There is a mild-moderate age-related decline in CCO oxidation rate (rate of change in concentration of oxidized cytochrome-c-oxidase (Δ[oxCCO]) in dark (Pearson correlation coefficient R = −4 and coefficient p value, p = 0.03). (**b**) Rate of hemoglobin oxygenation (rate of change in hemoglobin difference (Δ[HbDiff])) has a mild positive association with age which is not statistically significant (Pearson correlation coefficient R = 0.3 and coefficient p value, p = 0.06).

Age-dependency of mitochondrial oxidation rate as well as retinal oxygenation rate during dark adaptation are illustrated in Fig. 2 where data from individuals are shown by age. There is a moderate negative association between mitochondrial oxidation rate (at 15 min) with the age of the subjects (Fig. 2a). The association with hemoglobin difference (retinal oxygenation) is weaker with age and mirrors that of oxCCO (Fig. 2b).

**Figure 3:**
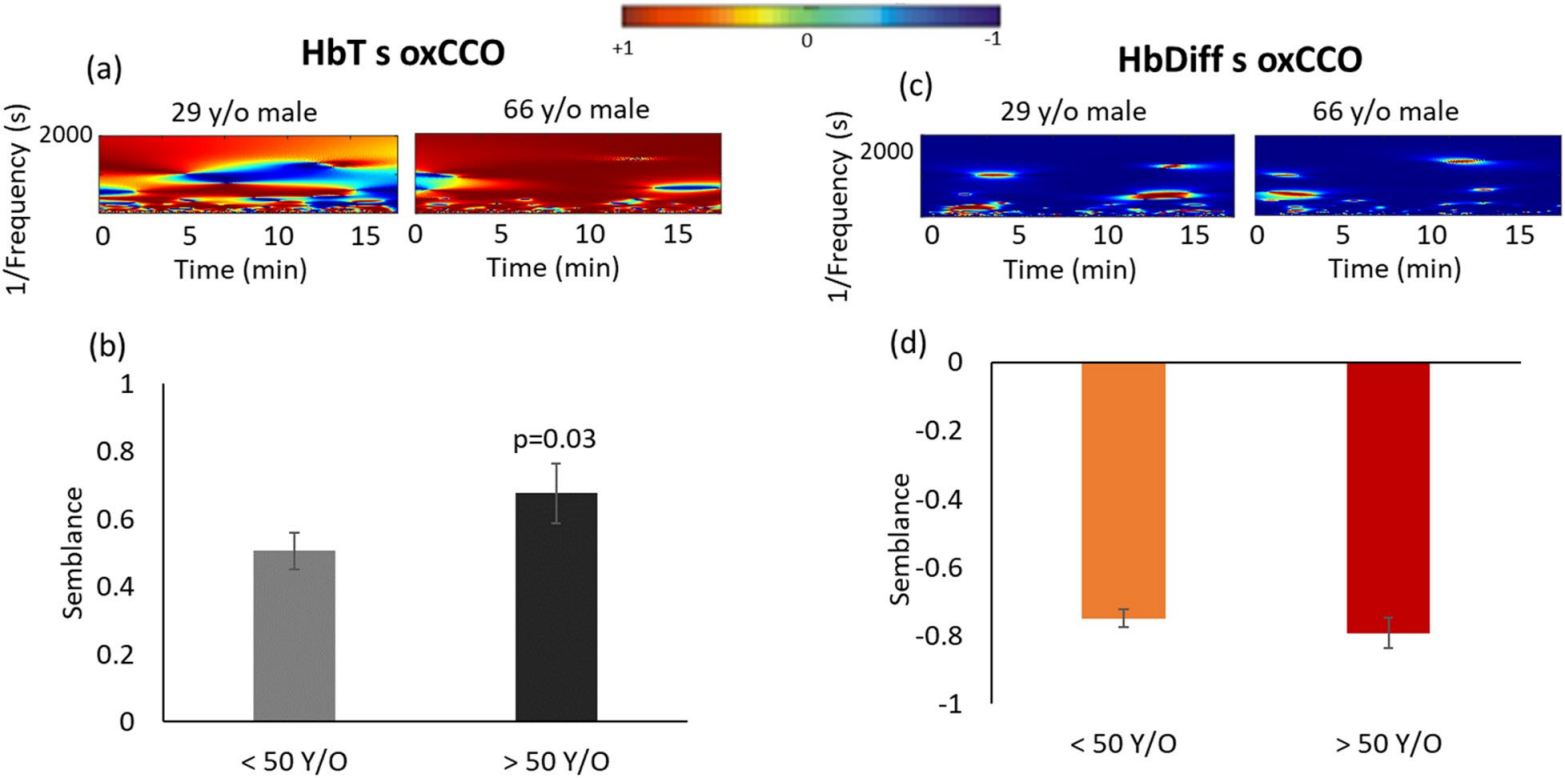
Semblance between mitochondrial oxidative metabolism (oxCCO) with total and difference hemoglobin signals (HbT and HbDiff markers for retinal blood volume and oxygenation, respectively) in dark. (**a**) HbT s oxCCO is greater in the 66-year-old (y/o) male compared to the 29 y/o male shown by predominantly red color representative of semblance + 1 ~ zero phase difference across all frequencies over time. (**b**) Group data for HbT s oxCCO averaged over all frequencies also showing significantly stronger semblance between total retinal blood volume with mitochondrial metabolism in subjects > 50 y/o (p = 0.03). (**c**) Showing a consistently antiphase relationship between HbDiff and oxCCO signals in dark in representatives 29 and 66 y/o male subjects (HbDiff s oxCCO predominantly blue across all frequencies, semblance −1 ~ 180° phase difference in both subjects). (**d**) Group data averaged across all frequencies showing no difference in HbDiff s oxCCO between < 50 and > 50 y/o, signifying a strong decoupling between retinal oxygenation and mitochondrial metabolism during dark adaptation across all ages. The color maps are produced using a MATLAB program, comparing time series using Semblance Analysis, version 1.0.0.0, found at https://uk.mathworks.com/matlabcentral/fileexchange/18409-comparing-time-series-using-semblance-analysis.

Semblance between total blood volume (HbT) and retinal oxygenation (HbDiff) with mitochondrial oxidative metabolism (oxCCO) are demonstrated in Fig. 3. Semblance is the cosine of phase difference between two signals in time–frequency domain. The color maps in Fig. 3a show the semblance between HbT and oxCCO signals during dark adaptation in a representative 29- and a 70-year-old male, respectively. In the younger subject, HbT has a varying phase association with oxCCO during dark adaptation (Fig. 3a), as the color map for HbT s oxCCO varies between red and blue colors across all frequencies over time (red color corresponding to 0-degree phase difference or + 1 semblance and blue corresponding to 180-degree phase difference or − 1 semblance). However, in the older subject, oxCCO is more synchronous with HbT (predominantly red). Group data shown on Fig. 3b represent the mean semblance between HbT and oxCCO averaged across all frequencies, which is significantly greater in the older subjects (0.7 ± 0.09) than the younger ones (0.5 ± 0.06), p = 0.03.

Figure 3c represents color maps for HbDiff and oxCCO semblance (HbDiff s oxCCO) in a representative 29-and a 66-year-old male, respectively. In both subjects, the oxCCO signal (or mitochondrial oxidative metabolism) has a perfectly antiphase relationship with the HbDiff signal (or choroidal oxygenation) across all frequencies and over time, which is shown by a consistent blue color signifying a semblance of −1 in Fig. 3c. This means any reduction or increase in retinal oxygenation is due to variations in mitochondrial oxygen uptake, rather than a change in blood flow. Figure 3d represents the average data across all frequencies in all subjects separated by age (< 50 and > 50 years old). It shows a strong negative semblance corresponding to a consistent antiphase relationship between retinal oxygenation and mitochondrial metabolism in dark in both young and old group (HbDiff s oxCCO: −0.7 ± 0.06 and −0.8 ± 0.05, respectively).

## Discussion

In this study, real time physiological changes in retinal respiration were assessed optically for the first time during early stages of dark adaptation. Our technology (bNIRS) provides a rapid in vivo assessment of the dynamics of mitochondrial oxidative metabolism (oxCCO) as well as dynamics of retinal oxygenation (HbDiff) and hemodynamics (HbT) using reflected light from the retina. The data do not provide steady state readouts, only relative changes. Our results show that during dark adaptation, when the retina is challenged metabolically, there are significant changes in both retinal mitochondrial function and hemodynamics with age. Our measurements were carried out in complete darkness, and while part of the light source used to probe the retina (> 710 nm) was dim deep red and just within the spectral sensitivity of human cones, it does not stimulate the rod population whose peak spectral sensitivity is around 495 nm^26^. Hence this would have no effect on dark adaptation.

Subjects were asked to place the beam from the light source in their central visual field. The collecting fibre was directly above the source with both targeting the macular. The source light appeared as a clear small target centrally. Subjects reported no light perception outside this region even at the end of the experiment when they were relatively well dark adapted. Hence, there was minimal light scatter. Even if there were undetectable illumination outside the macular, it would be very unlikely to result in a detectable back reflection. Our recordings targeted the macular region that spanned peaks in both rod and cone density. Saccadic eye movements during recordings would have increased the region of the central retina that was sampled.

To date, our technology is unable to resolve signals from individual retinal layers. But in any path through retinal depth, the greatest concentration of mitochondria will be found tightly packed in photoreceptor inner segments, and the macular has the greatest photoreceptor concentration. While our mitochondrial measurements summed signals across retinal depth, those from photoreceptors must have been paramount and we believe that they gave rise to the main mitochondrial signal. However, signal isolation over depth has to be a key target for future research perhaps similar to Chong et al.^27^.

As the macular was targeted in the experiments, the blood measurements here would have primarily sampled the choroidal blood supply as the macular region has few retinal vessels but the largest choroid blood supply. Taken together, these points reinforce that we were measuring mitochondrial responses in macular photoreceptors and their choroidal blood supply. However, although we argue that these were the primary signal sources, they undoubtedly contained smaller signals from other sources. Further work is needed by adopting Monte Carlo light diffusion computational techniques to estimate and quantify the photon distributions and pathlengths across and within the retina.

Dark adaptation imposes significant metabolic stress on the outer retina and the younger retinae (under 50 years old) respond to this by increasing the rate of oxidative metabolism to meet the increased ATP demand^2,4,28,29^. The rate of mitochondrial oxidation in this group was significantly faster than that in those older than 50 years old from 3 min onward. This was shown by a greater increase in their oxCCO signal per minute, which persisted for the 15 min measurement duration. In the older retinae, mitochondrial oxidation showed little change in response to the initial darkness and declined gradually over the measuring period of 15 min.

The drop in the oxCCO signal in the older group as the measurement progressed could be due to decreased retinal energy state as the older retinae are probably incapable of efficiently meeting the high ATP demand in darkness. The slower rate of oxidative metabolism in this group, which was significantly different from the younger group as early as 3 min into darkness, also contributes to the oxCCO depletion during prolonged stress in dark. It is possible that the older retinae at this point are more reliant on glycolysis for ATP production, although this is a less efficient route of energy production. In support of this, it has been shown in primate retinae that glycolysis increases in ageing with a significant drop in the activity of cytochrome-c-oxidase^30^. The less efficient production of ATP via glycolysis finds some harmony with the slower rate of oxidative metabolism with age that we reveal.

The decline in CCO oxidation with age could partly be attributed to the smaller number of rods in the older group. In support of this, it has been shown that rod density measured at the annular region of an eccentricity of 5 to 8 mm drops linearly with age^31^. However, these data are subjected to a great amount of noise with some individuals in their 60s having the same number of rods as those in their 20s. This high variability is also seen in our data as in Fig. 2a, where rate of change in oxCCO is similar for a 21- and a 66-year-old subject.

We demonstrated that changes in choroidal oxygenation (HbDiff) mirrored those in mitochondrial oxidative metabolism (oxCCO). The fact that the increase in oxCCO aligned with a drop in HbDiff is indicative of enhanced oxygen uptake by the retina to facilitate oxidative metabolism. Similarly, a drop in oxCCO in the older subjects during dark being in line with the increase in HbDiff implies that oxidative metabolism is strictly inhibited in the older retinae over time leading to an increase in choroidal oxygenation.

Significant changes found in choroidal oxygenation (HbDiff) occur later than those in mitochondrial oxidation (oxCCO), which is consistent with the idea that changes in blood oxygenation are driven by mitochondrial demand. Studies on retinal blood flow changes during dark adaptation also reveal a persistent 40–70% increase after transition from light to dark in response to increase mitochondrial demand^32–34^. There may also be other contributing factors for the difference in oxCCO signal leading that for HbDiff between young and old groups. However, while weight of evidence points in this direction, it is not conclusive as very small early undetected changes in oxygenation may impact may guide subsequent events. Generally, a bottleneck in delivering substrate in the oxCCO signal will manifest itself as oxidation of CCO if oxygen supply does not change. Alternatively, increased oxygen supply in the presence of adequate substrate supply will also manifest as an oxidation^14^. In light of this problem, it is important to highlight the key roles that mitochondria play not only in metabolism but also ageing. There may be a range in which reduced oxygenation does not impact on mitochondrial function. Tiede et al.^35^ provide data showing that mitochondrial membrane potential can actually be enhanced by reducing oxygen levels. Further it is important to remember that oxygen is also the essential substrate for formation of reactive oxygen species which drives ageing. Taken together there is much that we need to know to unravel the relationship between mitochondria, oxygen, metabolism and ageing. This needs to be placed firmly among the many other feature of ageing that centre upon mitochondria including mitochondrial DNA mutation accumulation, their patterns of fusion and fission, and the often-dual role that they play where in one situation when their production of reactive oxygen species can be protective and in others when it can drive inflammation^7^. To complicate matters further, mitochondrial function shifts during the day^36^ as may their relationship with oxygen.

The strong negative semblance or antiphase synchrony between HbDiff and oxCCO also implies that those changes in choroidal oxygenation are likely to be the result of shifts in mitochondrial O_2_ uptake, due to retina’s high oxygen demand in dark^37^. In older subjects there is also a significantly stronger coupling between total blood volume and mitochondrial metabolism (HbT s oxCCO), which is not seen in the younger group. It may be possible that mitochondrial activity needs to be more strongly regulated by blood volume in the aged retina due to reduced spare capacity. Therefore, there may be greater need to tighten the relationship between blood and mitochondria in the older subjects. Furthermore, retinal blood flow is reduced with age^38,39^ and the ability of the blood to communicate with mitochondria is also reduced due to thickening of the Bruch’s membrane^40,41^. This requires increased coupling between mitochondrial metabolism with hemodynamics during aged decline. It is unclear how age-related changes to the cellular architecture of the retina map onto our metabolic data. However, our data are based on moment-by-moment dynamic events which are independent of the architectural changes and differences between the absolute number of cells found in the two age groups.

While it is known that the dark adaptation function measured by rod intercept time is delayed with age^18–20,42^, the extent of the difference between the two age groups in terms of the mitochondrial response in the first 3 min is unexpected. It would appear that mitochondria in the ageing group failed to show any increase in metabolism when challenged. Moreover, the gradual rise in the HbDiff signal over time seen in older subjects may be due to reduced mitochondrial demand and also a shifting towards glycolysis. Reduced rod numbers with age may also play a role^31^. Further, age-related thickening of Bruch’s membrane^41,43^, limiting oxygen penetration is likely to be a contributory factor. It is equally possible that this results from a combination of these factors.

Declining visual function in an ageing human population is a major challenge and identifying when normal ageing is transformed into disease is critical for early interventions to save sight. This study has shown that mitochondrial decline in the ageing outer retina and its choroidal blood supply can be measured with NIRS. Full dark adaptation, particularly in older subjects can take > 40 min^42,44^, but our analysis of mitochondrial changes, which were centred on the macular region, reveals statistically significant age-related differences within the first 3 min of adaptation. This represents a significant advance in terms of our ability to assess key physiological markers in individuals.

Our method is non-contact, and with no restrictions on eye movements. This undoubtedly adds noise to our data, but potentially provides wider application of the technology. To minimise variability, subjects’ pupils were artificially dilated, but it is also possible to record retinal metabolic changes in dark environment without dilation, which adds further applicability to the technology. However, the data produced without standardised dilation have reduced clarity due to increased noise. (Data are not reported here).

The bNIRS algorithm uses the difference between the absorption spectra of oxidised minus reduced CCO to estimate changes in the redox state of mitochondrial proteins. However, the interpretation of the change in mitochondrial oxidation through oxCCO measurement is complex, as it represents a change in equilibrium. This change could be due to variations in any of multiple factors and the balance between them including oxygen supply, glucose or other substrate supply, pH, temperature, etc. Therefore, understanding the oxCCO signal is sometimes challenging due to the many factors that can cause increased oxidation or reduction of the enzyme. This complexity could be resolved through computer modelling of the physiology^14^. Another limitation of the technique which could also be dealt with using computer modelling is the estimation of the currently unknown optical pathlength of light in our measurement.

One factor that we have not considered is the potential switch between mitochondrial function and glycolysis. It is perfectly possible that glycolysis picks up when mitochondria are eroded by ageing. There is evidence from the mouse model that glycolysis plays a role in the maintenance of high metabolic activity in the retina^45^. However, there are fundamental differences between mouse and human retinae and sampling the mouse retina optically with our technology is demanding due to the small eye size. In fact, our research has been greatly simplified by the transition to human not only for size consideration but also for avoidance of anaesthetics, the potential to sample different areas as well as the relevance to the human condition.

The process of dark adaptation is disrupted in AMD^19,46^, a disease of ageing which has an association with mitochondrial dysfunction. Animal models of AMD have clear mitochondrial abnormalities from early retinal differentiation onward^47^, while RPE cells collected from AMD patients have mitochondrial respiratory abnormalities^48^. Given our results, it may be possible to rapidly assess subjects at risk of AMD and other metabolic retinal diseases using initial dark adaptation shifts long before disease is apparent.

## Supplementary Information

Supplementary Information.

## Data Availability

The data that support the findings of this study are available on request from the corresponding author GJ. The data are not publicly available due to them containing information that could compromise research participants privacy.
