# Supplementary material for: Watching the human retina breath in real time and the slowing of mitochondrial respiration with age
Source: Sci Rep. 2023 Apr 20;13:6445. doi: 10.1038/s41598-023-32897-7 (PMC10119193; doi:10.1038/s41598-023-32897-7)
Supplement: Supplementary file 1 — Supplementary Information. [file 41598_2023_32897_MOESM1_ESM.docx]

**Supplementary Information**

Fig. 1 illustrates the residual analysis to investigate crosstalk in the bNIRS oxCCO measurement. Measured attenuation spectrum is compared against back-calculated attenuations from 2- and 3-chromophore fit in Fig. 1a. The back-calculated attenuation from 3-chromophore fit (red) matches the measured attenuation (black) whilst the back-calculated attenuation when solving only for 2 chromophores (blue) does not fit the measured attenuation. Further, the residual error from 2-chromophore fit in all the subjects (across 780-900 nm), have a well-defined shape that matches the extinction coefficient of oxCCO between 780-900 nm (Fig. 1b, blue spectrum). This shows when solving only for HHb and HbO_2_, the oxCCO chromophore remains unaccounted for in the field of view. However, when solving for 3 chromophores (including oxCCO), the residual error has no shape and randomly fluctuates around zero (Fig. 1b, red spectrum). This demonstrates that all the chromophores in the field of view are accounted for when oxCCO extinction coefficient is included in the measurement algorithm. This analysis proves that the oxCCO signal measured this way is a true representative of changes in oxidised-CCO and not a crosstalk artifact from the haemoglobin signal.


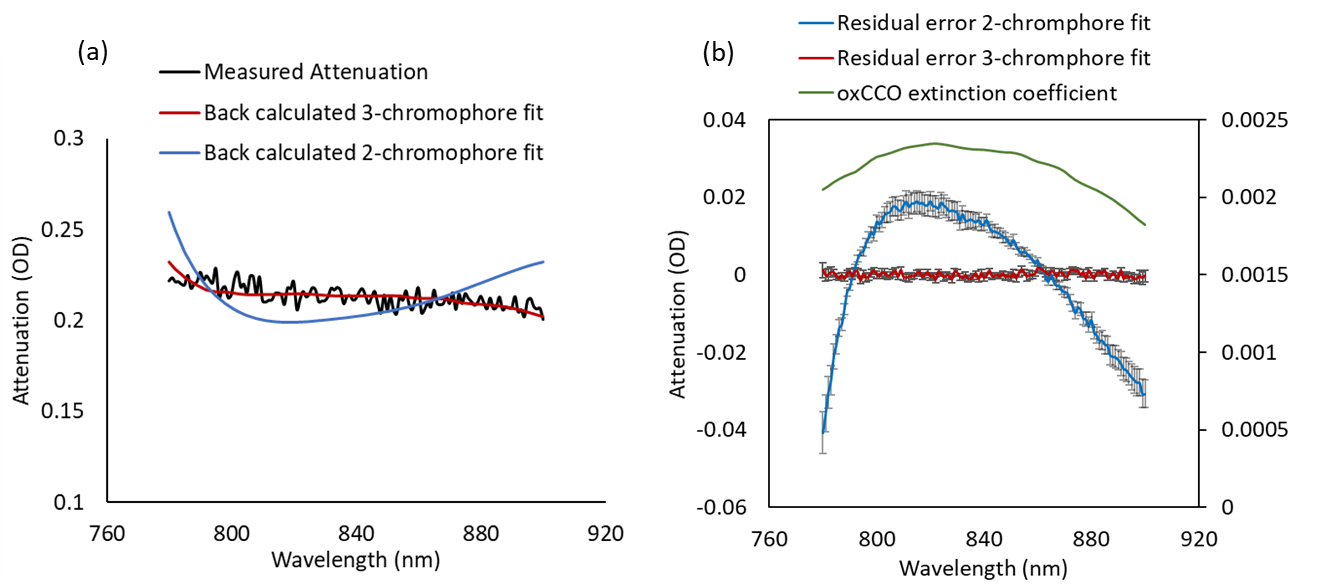


**Figure 1:** (a) Example attenuation spectrum estimated from measured intensity change (black line), back-calculated attenuation estimated using a 3-chromophore fit accounting for HHb, HbO_2_ and oxCCO (red line), back-calculated attenuation estimated only accounting for HHb and HbO_2_ (blue curve). (b) Residual errors from 3- and 2-chromophore fit against the extinction coefficient of oxCCO. The residual spectrum from fitting only HHb and HbO_2_ in the measurement algorithm from the real attenuation spectrum have the same shape as oxCCO absorption spectrum (blue), which suggests that a chromophore with a similar shape to oxCCO is needed to fully explain the spectra. The residual error from 3-chromophore fit (red) has no shape and randomly oscillates around zero. The residual errors from 2- and 3-chromophore fit are averaged spectra for all the subjects and error bars are standard errors from the mean.
